# Lossless Contrast Enhancement of Color Images with Reversible Data Hiding

**DOI:** 10.3390/e21090910

**Published:** 2019-09-18

**Authors:** Hao-Tian Wu, Yue Wu, Zhihao Guan, Yiu-ming Cheung

**Affiliations:** 1 School of Computer Science and Engineering, South China University of Technology, Guangzhou 510006, China; 201664866012@mail.scut.edu.cn (Y.W.); 201821033825@mail.scut.edu.cn (Z.G.); 2 Department of Computer Science, Hong Kong Baptist University, Hong Kong, China; ymc@comp.hkbu.edu.hk

**Keywords:** contrast enhancement, reversible data hiding, HSV color model, color difference, entropy

## Abstract

Recently, lossless contrast enhancement (CE) has been proposed so that a contrast-changed image can be converted to its original version by maintaining information entropy in it. As most of the lossless CE methods are proposed for grayscale images, artifacts are probably introduced after directly applying them to color images. For instance, color distortions may be caused after CE is separately conducted in each channel of the RGB (red, green, and blue) model. To cope with this issue, a new scheme is proposed based on the HSV (hue, saturation, and value) color model. Specifically, both hue and saturation components are kept unchanged while only the value component is modified. More precisely, the ratios between the RGB components are maintained while a reversible data hiding method is applied to the value component to achieve CE effects. The experimental results clearly show CE effects obtained with the proposed scheme, while the original color images can be perfectly recovered. Several metrics including image entropy were adopted to measure the changes made in CE procedure, while the performances were compared with those of one existing scheme. The evaluation results demonstrate that better image quality and increased information entropy can be simultaneously achieved with our proposed scheme.

## 1. Introduction

Contrast enhancement (CE) is one important topic in image processing. It has been widely applied to medical and industrial images, which helps to improve the visibility of content details [1]. Due to the limits in image acquisition, not all images have satisfactory quality and contrast effects, the CE technique is usually used to improve understandability and readability of images. Normally, the contrast-enhanced images with better quality can be generated to show the interested details.

As quite a lot of CE methods have been proposed (e.g., [1,2,3,4]) to increase visibility of image content, part of information in the original image may be lost because the image content is permanently changed. In recent decades, the technique of reversible data hiding (RDH) (e.g., [5,6,7,8,9,10,11,12,13,14,15,16,17,18]) has been developed to carry extra information in a lossless manner by exploiting the redundancy in data representation (e.g., [19]). Recently, lossless CE has been proposed so that a contrast-changed image can be converted to its original version by maintaining information entropy in it. Specifically, CE effects can be obtained with RDH such as in [20,21,22,23,24,25,26,27,28,29] so that an original image can be recovered from its contrast-enhanced version. Moreover, extra data can be hidden into the contrast-enhanced images to facilitate other functionalities such as authentication and content annotation.

Although several lossless CE methods have been proposed, only a few of them are applicable to color images. Presently, color images are popularly used and transmitted because a color image is a powerful visual descriptor and contains more information than a grayscale image [8]. Therefore, there exists a large potential to perform lossless CE on color images. For instance, many medical and industrial images in the daily use are in color, while any information loss in them is undesired. Thus, it is advantageous to perform lossless CE on them in the sensitive applications.

The main difference between color and grayscale images is that the pixels in a color image consist of three components (e.g., red, green and blue), while a pixel value in a grayscale image is a single number representing the brightness. For a grayscale image, CE is performed to adjust image brightness, which visually enriches the lays of graphics and avoids the image from being too dark or light. As for color images, the procedure of CE is more complicated since a color image can be represented by a three-dimensional matrix, and there are high correlations between the three components.

To achieve lossless CE of color images, it is straightforward to apply the methods initially proposed for grayscale images to each channel of a color image. In other words, every channel of an RGB image is considered to be a single-valued image and is separately enhanced by applying the existing CE methods. Then a new color image is formed by combining the contrast-enhanced image in every channel. However, directly applying the existing methods to each channel may cause serious color distortions because each channel is separately processed. An improved scheme was proposed in [28] to ensure uniform enhancement of each channel, which is called uniform contrast enhancement (UCE) of RGB channels. Specifically, the same number of histogram bins are used for data embedding in each of the three channels. Nevertheless, visual distortions may still be caused after applying the UCE scheme when more histogram bins are modified.

Under these circumstances, a new image CE scheme with RDH is proposed based on the HSV (hue, saturation, value) color model. In HSV color model, color images can be processed according to human vision system so that color distortions caused by CE can be avoided. To perform image CE in HSV model, the relation between the RGB model and the HSV model should be used. Specifically, the RGB channels are converted to the Max, Median and Min channels according to the numerical values of red, green and blue (Max channel consists of the largest value in every pixel, Median channel consists of the median value in every pixel, while Min channel consists of the smallest value in every pixel). The Max channel (i.e., the value channel in HSV model) is directly enhanced by applying the existing lossless CE method (e.g., [27]). The Median and Min are also modified to maintain the ratios of between them and the Max component in each pixel, respectively. In this way, the hue and saturation components in every pixel are perfectly maintained so that no color distortion will be caused.

To validate the efficacy of the proposed scheme, two sets of color images were used, and the experimental results show that satisfactory CE effects can be achieved. As visual distortions were caused with the UCE scheme in some cases, there was no color distortion introduced by applying the proposed scheme. Although truncation errors may be introduced in maintaining the hue and saturation components in every pixel, the value component (i.e., the Max channel) can be exactly recovered. In addition, the experimental results show that there was little difference between the recovered images and the original ones so that perfect recovery of the original images can be achieved. Moreover, the evaluation results demonstrate that better image quality and increased information entropy can be simultaneously achieved with our proposed scheme.

The rest of this paper is organized as follows. In the next section, several schemes used for reversible CE of grayscale images will be briefly reviewed. Then a new CE scheme with RDH will be proposed for color images in Section 3. In Section 4, the experimental results of the proposed scheme will be presented, including the comparison with the UCE scheme in [28]. Finally, a conclusion will be drawn in Section 5.

## 2. Reversible Contrast Enhancement Methods for Grayscale Images

In this section, the reversible image data hiding method with CE proposed in [20] will be first introduced, followed by the improved preprocessing in [27] and the automatic CE method with RDH in [23,28].

### 2.1. RDH with Image Contrast Enhancement

To the best of our knowledge, the first attempt to achieve CE effect by RDH was made in [20], where a procedure named histogram bin expansion is proposed. First, a histogram is generated by counting the number of each pixel value in a grayscale image. Then the highest two bins in the obtained histogram are chosen, which are denoted as $f_{L}$ and $f_{R}$ ($f_{L}<f_{R}$). The chosen bins ($f_{L}$ and $f_{R}$) are expanded into two adjacent bins, respectively. By scanning every pixel in the image, a pixel value *f* is modified to $f^{\prime }$ by

(1) $$f^{\prime }=\left\{\begin{array}{cc} \;f-1, & \mathrm{if}\;f<f_{L}\\ \;f, & \mathrm{if}\;f_{L}<f<f_{R}\\ \;f+1, & \mathrm{if}\;f>f_{R} \end{array}\right.$$

or

(2) $$f^{\prime }=\left\{\begin{array}{cc} \;f-b_{i}, & \mathrm{if}\;f=f_{L}\\ \;f+b_{i}, & \mathrm{if}\;f=f_{R} \end{array}\right.,$$

where $b_{i}$ is the *i*-th bit value (0 or 1) to be hidden given that *f* is the *i*-th pixel value contained in $\left\{f_{L},f_{R}\right\}$. After applying Equation (1) or Equation (2) to every pixel in the whole image, the histogram is modified and the highest two bins in the newly generated histogram are further chosen to be expanded. By repeatedly conducting histogram bin expansion, the effect of histogram equalization can be achieved with data embedding.

To avoid the overflow and underflow of pixel values that may be caused after applying Equation (1), preprocessing is conducted in the method proposed in [20]. Suppose that S pairs of histogram bins are expanded in total. The bins with value from 0 to S−1 and from 256−S to 255 should be emptied. Specifically, the pixels values from 0 to S−1 are added by *S*, and the pixels values from 256−S to 255 are subtracted by *S*. To record the pixels modified, a binary location map with the same size as the image is generated and compressed with the JBIG2 standard [30] if needed.

To recover the original image, the information recorded in preprocessing and histogram bin expansion should be kept. In particular, the value of *S*, the location map generated in preprocessing, the least significant bits (LSB) of the last 16 pixels before the last time of histogram bin expansion, the values of previously expanded bins, are all embedded by performing histogram bin expansion for *S* times in total. Finally, a contrast-enhanced image is generated after the LSBs of the last 16 pixels are replaced with the values of the last two bins expanded for data embedding.

### 2.2. An Order-Preserving Preprocessing

One drawback of the method in [20] is that visual distortions may be introduced in preprocessing. After adding *S* to the pixel values from 0 to S−1 and subtracting *S* from the pixels values from 256−S to 255, the order of pixel values cannot be kept. To avoid the visual distortions especially for a large value of *S*, a new preprocessing strategy is proposed in [27] to preserve the order of pixel values. First, the empty bins are shifted to the two sides of the histogram. Then histogram bin merging is conducted if the number of empty bins on any side is less than *S*. Furthermore, it is required that only two adjacent bins are eligible to be merged to generate a new empty bin (which is to be shifted outside of the non-empty bins afterwards) while the merged bin cannot be merged any more. To record the changes made in preprocessing, bookkeeping information is generated instead of generating a location map as in [20]. Interested readers can refer to [27] for the details of implementation. Although satisfactory image quality can be achieved in [27] for grayscale images, directly applying the method to each channel of an RGB image may still cause visual distortions in the experiments.

### 2.3. Automatic Contrast Enhancement with Reversible Data Hiding

In [23], an automatic CE scheme with RDH is proposed by merging the lowest histogram bin with its adjacent one to create an empty bin. To reduce the over enhancement effects that may be caused by applying the algorithm in [23], an automatic brightness preserving CE method is introduced in [28]. To create an empty bin, a bin is merged with one of its adjacent bins given that the sum of the two bin heights is the smallest in the histogram. After shifting those bins between the highest one and the merged bin, the highest bin can be expanded into two adjacent ones for data embedding. The procedure is repeatedly to achieve histogram equalization effect. In addition, the direction of histogram bin shifting is adjusted according to brightness of an original image at each repetition of data embedding. For example, if the brightness after data embedding is less than the original image, then the bins are shifted to increase the pixel values. Otherwise, bins are shifted to decrease the pixel values if the brightness after data embedding is more than the original one.

Similar to [27], side information needs to be recorded at each repetition to indicate the bin merging and histogram shifting. The procedure of data embedding is continued until the ever-increasing side information cannot be kept in the contrast-enhanced image. Consequently, CE with brightness preservation can be achieved with the method in [28]. However, directly applying it to each channel of the RGB model may cause visual distortions. To alleviate the distortions, an improved scheme was proposed for color images to ensure uniform enhancement of each channel, which is hereinafter called the UCE scheme. Specifically, the same number of histogram bins are used for data embedding in each of the three channels. Nevertheless, visual distortions may still be caused after applying the UCE scheme, especially when many histogram bins need to be expanded. To cope with this issue, the correlations between the three channels should be considered in the procedure of CE. As the HSV color model is defined according to human vision system, we will propose a new scheme based on it for CE of color images in the following section.

## 3. Proposed Scheme for Contrast Enhancement of Color Images

In this section, the HSV color model will be first introduced. Then a new CE scheme with RDH will be proposed based on it. The procedure of implementing the proposed scheme for CE of a color image and that of recovering the original image will be further provided, respectively.

### 3.1. HSV Color Model

The human visual system characterizes a color image by its brightness and chromaticity [31]. The chromaticity is represented by hue and saturation components in HSV color model, while the brightness corresponds directly to luminous intensity. So, there are three components in HSV model, i.e., hue (H), saturation (S), and brightness (V). Specifically, the HSV model is introduced in [32] by

(3) $$\begin{array}{cc} \; & H=\left\{\begin{array}{cc} \;60\times (\frac{G-B}{Max-Min}), & \mathrm{if}\;\;Max=R\\ \;60\times (\frac{B-R}{Max-Min}+2), & \mathrm{if}\;\;Max=G\\ \;60\times (\frac{R-G}{Max-Min}+4), & \mathrm{if}\;\;Max=B\\ \;not\;defined, & \mathrm{if}\;\;Max=0 \end{array}\right.\\ \; & S=\left\{\begin{array}{cc} \;\frac{Max-Min}{Max}, & \mathrm{if}\;\;Max\neq 0\\ \;0, & \mathrm{if}\;\;Max=0 \end{array}\right.\\ \; & V=\;Max \end{array},$$

where *R*, *G* and *B* represents the red, green and blue components of a pixel value, while Max and Min are the largest and smallest values of the three components, respectively. Please note that the values of *R*, *G* and *B* in Equation (3) range from 0 to 1. To ensure the H component is scaled to [0, 360], the H component is then processed by

(4) H=H+360,ifH<0.

To achieve CE effects, the V component in the HSV model can be processed by the methods proposed for grayscale images while the other two components are kept to avoid possible color distortions. Given that hue and saturation components are unchanged, no difference will be made in chromaticity. To avoid the color distortion in color images, we need to keep the H and S components unchanged. According to Equation (3), the ratios listed in Equations (5) and (6) need to be maintained so that the H and S components in the HSV model can be kept unchanged. Meanwhile, the V component is modified to achieve the CE effects by applying a scheme proposed for grayscale images (e.g., [27,28]).

Nevertheless, the gamut problem [33] may arise when transforming a color image from the RGB model to the HSV model. Please note that the V and S components in HSV model are represented by real numbers which range from 0 to 1, while the value in the H component is represented by an integer within the range of [0, 360]. In the following, we will introduce how to modify the *R*, *G* and *B* components to keep the H and S components in every pixel unchanged and achieve CE effects by modifying the V component.

### 3.2. Proposed Scheme

In the proposed scheme, the H and S components in every pixel are kept unchanged to avoid any color distortion. Meanwhile, the V component is modified to perform CE on an RGB image. To accomplish this in an RGB color image without color space transformation, the Max, Median and Min are first obtained from the red, green and blue components of a pixel value. Before performing CE on the Max channel, two ratios $c_{1}$ and $c_{2}$ are calculated from each pixel by

(5) $$c_{1}=\frac{Min}{Max}$$

and

(6) $$c_{2}=\frac{Median-Min}{Max-Min}.$$

By comparing Equation (3) with Equations (5) and (6), it can be seen that the H and S components can be kept unchanged by fixing $c_{1}$ and $c_{2}$ to ensure that no difference is made in chromaticity of a color image.

**Figure 1:**
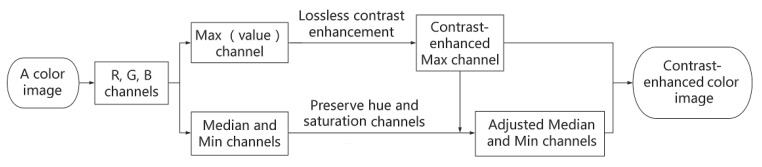
Proposed procedure for contrast enhancement of a color image.

To preserve image quality after CE, the reversible CE scheme in [27] is applied to the V channel in HSV model (i.e., Max channel). One advantage of applying CE on Max channel is that no overflow will be caused in the Min and Median to maintain the ratios of $c_{1}$ and $c_{2}$. Suppose that Max is modified to $Max^{\prime }$ after the CE process. According to Equation (5), Min is changed to $Min^{\prime }$ according to $Max^{\prime }$ and $c_{1}$ by

(7) $$Min^{\prime }=Max^{\prime }\cdot c_{1},$$

and the obtained $Min^{\prime }$ needs to be rounded to the nearest integer. According to Equations (5) and (6), Median is changed to $Median^{\prime }$ with $Max^{\prime }$, $c_{1}$ and $c_{2}$ by

(8) $$Median^{\prime }=Max^{\prime }\cdot \left(c_{2}-c_{1}\cdot c_{2}+c_{1}\right),$$

and the obtained $Median^{\prime }$ is also rounded to the nearest integer. The proposed procedure for CE of an RGB image is demonstrated in Figure 1, while the implementation details are summarized in Algorithm 1.
**Algorithm 1: Enhancing an RGB image based on HSV color model**
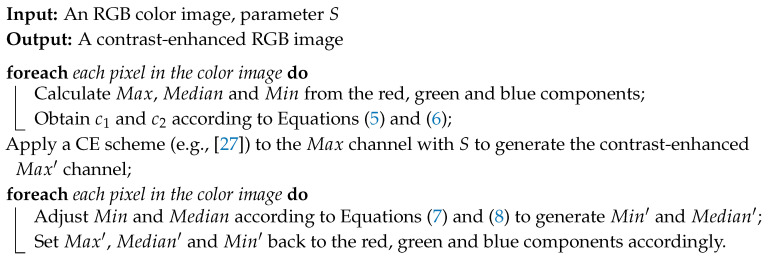


The phase of applying the scheme in [27] to the V (i.e., Max) channel includes the following steps:

(1) Calculate the histogram of Max channel and carry out the preprocessing described in Section 2.2. Then the bookkeeping data is generated to record the changes made in preprocessing.
(2) Find the highest two bins in the histogram and apply Equation (1) or Equation (2) to every Max value, respectively. Then find out the highest two bins in the newly generated histogram and repeatedly apply Equation (1) or Equation (2) until S−1 pairs of histogram bins have been expanded in total. Hide the necessary information during histogram bin expansion, including the bookkeeping data and its length.
(3) Collect the LSBs of the last 16 Max values, then apply Equation (1) or Equation (2) to the histogram excluding the last 16 Max values to embed value of *S*, the values of the previously expanded bins and those of the 16 original LSBs.
(4) Replace the LSBs of the last 16 Max values with the lastly expanded bin values so that the modified V channel (i.e., $Max^{\prime }$ channel) is generated.

### 3.3. Data Extraction and Image Recovery

**Figure 2:**
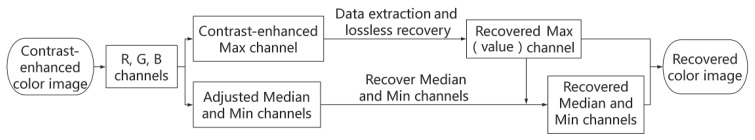
Proposed procedure of recovering the original color image.

The procedure of data extraction and recovering the original color image is described in Figure 2, while the implementation details are summarized in Algorithm 2.
**Algorithm 2: Recovering the original color image**
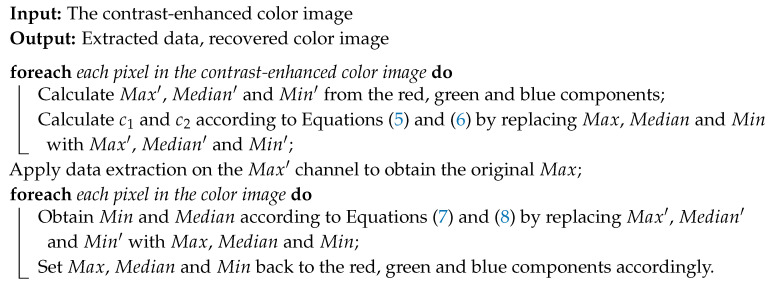


Within Algorithm 2, the phase of data extraction from the $Max^{\prime }$ channel and recovering the original one includes the following steps:

(1) Collect the LSBs of the last 16 values in the $Max^{\prime }$ channel and the last expanded two bins can be obtained as $\left\{f_{LL},f_{LR}\right\}$.
(2) Calculate the histogram of $Max^{\prime }$ channel except the last 16 values and then perform data extraction by
  (9) $$b^{\prime }=\left\{\begin{array}{cc} 1, & \mathrm{if}\;f^{\prime }=f_{LL}-1\;\mathrm{or}\;f^{\prime }=f_{LR}+1\\ 0, & \mathrm{if}\;f^{\prime }=f_{LL}\;\mathrm{or}\;f^{\prime }=f_{LR}\\ null, & otherwise \end{array},\right.$$
  where $f^{\prime }$ is a value in the Max channel and $b^{\prime }$ is the bit value extracted from $f^{\prime }$. In addition, the histogram is restored by changing $f^{\prime }$ to
  (10) $$f=\left\{\begin{array}{cc} f^{\prime }+1, & \mathrm{if}\;f^{\prime }<f_{LL}\\ f^{\prime }, & \mathrm{if}\;f_{LL}-1<f^{\prime }<f_{LR}+1\\ f^{\prime }-1, & \mathrm{if}\;f^{\prime }>f_{LR} \end{array}.\right.$$
(3) Obtain the value of *S*, the previously expanded bins and the original LSBs of the last 16 Max values from the extracted data. Then write back the values of the original 16 LSBs back.
(4) Calculate the histogram of the Max channel including the last 16 components. Then apply Equations (9) and (10) repeatedly for S−1 times to restore the Max channel after preprocessing.
(5) Obtain the bookkeeping data from the extracted data and recover the original Max channel with it.
(6) Make use of the rest extracted data (optional).

## 4. Experimental Results

In the experiments, six color images with the size of 512×512 downloaded from the USC website (http://sipi.usc.edu/database/database.php?volume=misc) and 24 images with the size of 768×512 downloaded from Kodak Lossless True Color Image Suite (http://www.r0k.us/graphics/kodak/) were used to evaluate the performance of the proposed method. Besides the auxiliary data such as bookkeeping information generated from a specific image to be enhanced, other data to be hidden into contrast-enhanced images consisted of a close number of 0 s and 1 s.

**Figure 3:**
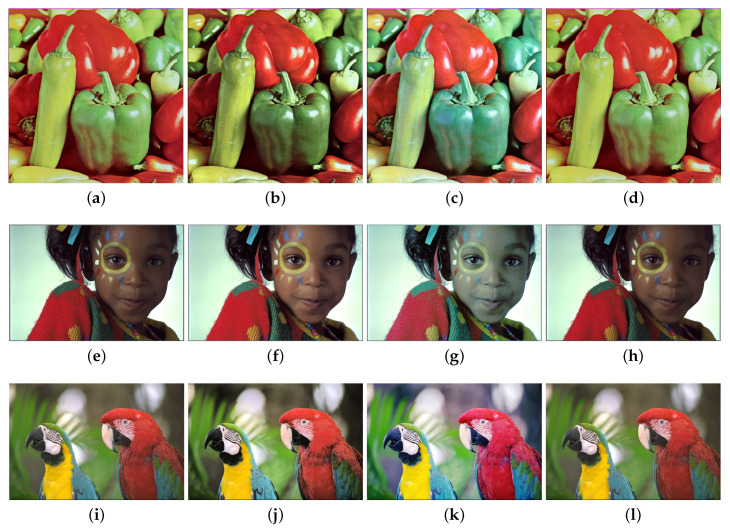
Contrast enhancement on three Kodak images by applying the proposed and the UCE scheme [28] with *S* = 50, respectively. (**a**) Original image “Peppers”, CIEDE = 0, Entropy = 7.298; (**b**) With proposed scheme, CIEDE = 8.98, Entropy = 7.537; (**c**) With the UCE scheme, CIEDE = 11.02, Entropy = 7.789; (**d**) Recovered (proposed), CIEDE = 0.09, Entropy = 7.279; (**e**) Original Kodak image, CIEDE = 0, Entropy = 7.293; (**f**) With proposed scheme, CIEDE = 4.30, Entropy = 7.496; (**g**) With the UCE scheme, CIEDE = 13.71, Entropy = 7.645; (**h**) Recovered (proposed), CIEDE = 0.03, Entropy = 7.295; (**i**) Original Kodak image, CIEDE = 0, Entropy = 7.368; (**j**) With proposed scheme, CIEDE = 6.10, Entropy = 7.642; (**k**) With the UCE scheme, CIEDE = 14.00, Entropy = 7.815; (**l**) Recovered (proposed), CIEDE = 0.13, Entropy = 7.365.

To fairly compare the performance of the proposed scheme with the UCE scheme in [28], the reversible CE method in [27] was adopted in the two schemes, respectively. In implementing the UCE scheme, the reversible CE method was applied to every channel in RGB color model by expanding the same number of histogram bins. In implementing the proposed scheme, the reversible CE method was only applied to the V channel (i.e., Max component in every pixel), while the Min and Median components in every pixel were adjusted to maintain the hue and saturation channels in HSV color model. For every test image, parameter *S* was chosen from 1 to more than 40 to apply the method in [27]. For part of test images, parameter *S* could be set more than 50 to increase the CE effects. Three color images and their contrast-enhanced images generated by applying the two schemes with *S* = 50 are shown in Figure 3, respectively.

### 4.1. Color Difference and Image Entropy

To measure the color difference between the original and contrast-enhanced images, a metric called CIEDE2000 [34] was adopted, which was defined by the International Commission on Illumination to quantify difference between two colors. A higher value of CIEDE2000 means larger difference while CIEDE2000 = 0 when there is no difference between two colors. From the contrast-enhanced images as shown in Figure 3, it can be seen that more color differences were caused by applying the UCE scheme. Specifically, the girl’s face in Figure 3g became a little greener, but there is no such color distortion in Figure 3f after applying the proposed scheme. Similarly, the colors of original images were better preserved by the proposed scheme, as shown in Figure 3b,j. For each contrast-enhanced image, the value of CIEDE2000 was calculated and labelled below it (denoted by CIEDE for short). Although CE effects were achieved with the UCE scheme, it can be seen from the obtained CIEDE2000 values that more color differences were introduced than using the proposed scheme.

In addition to color difference between the original and contrast-enhanced images, entropy of each image was further calculated to measure information in it. Given that the proportion of those pixels with value *i* in a grayscale image is $p_{i}$ ($0\leq p_{i}\leq 1$ and $\sum_{i=0}^{255}p_{i}=1$), the one-dimensional (1D) entropy of the grayscale image, which is denoted by *H*, is calculated by

(11) $$H=-\sum_{i=0}^{255}p_{i}\times \log p_{i}.$$

As *H* represents uncertainty or randomness in the clustering features of the gray distribution, the higher its value is, the more information exists in the image. For color images, we calculated the 1D entropy of each color channel (i.e., R, G, B) and then generated the average one as that of the whole image. The obtained entropy values are also labelled below the corresponding images as shown in Figure 3, respectively.

To further compare the performance of the UCE scheme and the proposed one, the two schemes were applied to all test images in the two sets by setting different values (20, 30 and 40) to parameter *S*, respectively. After calculating the metric of CIEDE2000 and entropy from every contrast-enhanced image, the mean values for every image set with the same *S* value are listed in Table 1, respectively. From the statistical results, it can be seen that color difference and image entropy were both increased after expanding more histogram bins for data embedding. For the same value of *S*, smaller mean value of CIEDE2000 was always obtained with the proposed scheme, indicating that less color difference was introduced than using the UCE scheme. The entropy obtained with the proposed scheme was smaller than that obtained with the UCE scheme, but apparently larger than that of the original image. Since larger color differences were made by applying the UCE scheme, part of the information gained with the UCE scheme was useless or unfavored. In other words, applying the proposed scheme for CE can increase the information in image content with less color difference with original images.

The entropy values of the three test images as shown in Figure 3 are also shown in Table 2, including the original images and the contrast-enhanced images after applying the UCE and the proposed scheme, respectively. It can be seen that the entropy values of the contrast-enhanced images were higher than those of the original images. As the three images enhanced with the UCE scheme had higher entropy values than those enhanced by the proposed scheme, color distortions were introduced by applying the UCE scheme. Applying the proposed scheme also increased the entropy values of the three images, but no visual or color distortion was caused.

To further compare the difference between the UCE and the proposed method, a *t*-test was conducted on the CIEDE2000 and entropy values obtained with the two methods. By setting *S* to 20, 30 and 40, the statistical results are listed in Table 3, respectively. It can be seen that the CIEDE2000 and entropy values were significantly different for the UCE and the proposed method, indicating that different color changes and information gain were made in the CE process. Actually, the information gain with the UCE scheme as listed in Table 1 was partly due to the color differences introduced in the CE process.

### 4.2. Contrast Enhancement Effects

To evaluate the CE effect, the relative mean brightness error (RMBE) and relative contrast error (RCE) defined in [35] were further calculated by comparing the contrast-enhanced images with the original ones. RCE is a value within [0,1] and RCE >0.5 when the contrast has been enhanced. RMBE <1 when the mean brightness has been changed, while RMBE =1 if the mean brightness is unchanged. Moreover, the Peaked Signal-to-Noise Ratio (PSNR) and Structural SIMilarity (SSIM) index [36] were calculated from the contrast-enhanced images according to the original images.

The evaluation results for the three images as shown in Figure 3 are given in Table 2. It can be seen that better image quality was achieved with the proposed scheme because higher SSIM and PSNR values were obtained. Since CE was performed on three channels of test images in the UCE scheme, the corresponding RCE values were higher than those obtained with the proposed scheme for two test images. For the color image as shown in Figure 3d, slightly higher RCE value was obtained with the proposed scheme than using the UCE scheme. Meanwhile, higher RMBE values were all obtained with the proposed scheme, indicating that the brightness of color images was better preserved.

In addition to the three test images as shown in Figure 3, the evaluation results were obtained from the 6 USC images and the 24 Kodak images, respectively. By setting *S* to 20, 30 and 40, the statistics (mean) of 6 or 24 images are shown in Table 4. As reflected by the mean RCE values, it can be seen that the effects of CE were significantly increased with *S*. Meanwhile, the decreases of the mean SSIM and PSNR values indicate that the differences between the contrast-enhanced and original images were enlarged after more histogram bins were expanded. Although the RCE values obtained by the UCE scheme [28] are higher than those obtained with the proposed scheme, generally higher PSNR, SSIM and RMBE values were obtained with the proposed method. In addition, the distortions caused by applying the UCE scheme also contributed to the increase of RCE, but there was no such distortion caused by applying the proposed scheme.

### 4.3. Original Image Recovery

With the proposed scheme, only the Value (i.e., Max) channel can be exactly recovered after extracting the data hidden in it. Although no overflow will be caused in adjusting the Median and Min components to keep the ratios unchanged, truncation errors may be caused so that exact recovery of the original image cannot be guaranteed. Nevertheless, image recovery was almost lossless because the differences between the original and recovered images were very small, which were measured by SSIM, PSNR, entropy and CIEDE2000 and the statistical results are shown in Table 5 for two image sets. It can be seen that the average SSIM values were all above 0.9988, the average PSNR were all above 54 dB, the differences in average entropy for two image sets were all no more than 0.004, and the average CIEDE2000 were all less than 0.120 for different values of *S* (i.e., 20, 30 and 40), showing that there were little differences between the original and the recovered images. Actually, it is difficult for human visual system to distinguish the recovered images from the original ones. Hence it can be said that the original images were perfectly recovered.

### 4.4. Pure Hiding Rate

**Figure 4:**
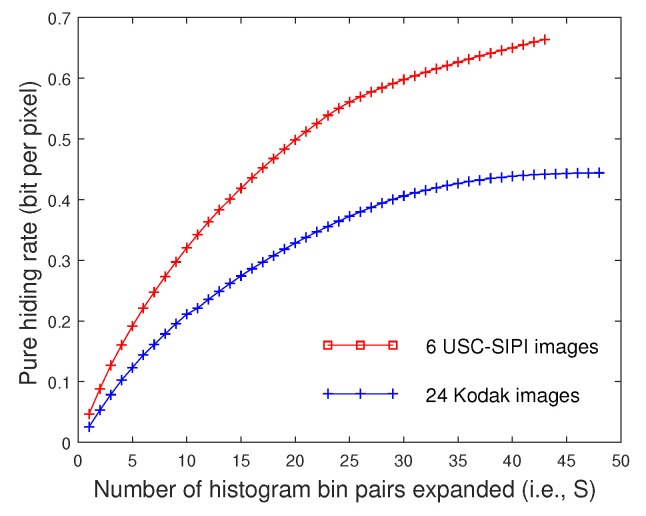
The average pure hiding rate of two image sets with respect to *S*.

For every image, the pure embedding capacity was calculated by subtracting the amount of side information from the total amount of embedded bits. The pure hiding rate was obtained after dividing the pure embedding capacity by the number of pixels, which is represented in bit per pixel (bpp for short). In Figure 4, the average pure hiding rates for 6 USC images and 24 Kodak images are plotted with respect to the parameter *S*, respectively. It can be seen that the pure hiding rate is increased by expanding more histogram bins for data embedding. For a large value of *S*, the curve of pure hiding rate turns to be stable for the amount of side information is also increased. The difference in pure hiding rate between the two image sets is due to different redundancy in image content. Though only the V channel was used for reversible data hiding, it can be seen that considerable amount of extra data could be hidden into the contrast-enhanced images.

## 5. Conclusions

In this paper, a new contrast enhancement scheme with reversible data hiding has been proposed for color images. Instead of separately applying the existing algorithm to each channel of the RGB color model, contrast enhancement is performed based on the HSV color model to avoid color distortion. Specifically, only the brightness component is modified while the chromaticity is well maintained by fixing the ratios between the RGB components in every pixel. The experimental results have demonstrated that better image quality can be obtained with the proposed scheme by comparing with the scheme proposed in [28] while achieving increased information entropy. In addition to perfect image recovery, extra data can be hidden into the contrast-enhanced images to enable other functionalities. The main contribution of this work is to significantly reduce the color distortions that may be introduced into the contrast-enhanced images by the existing CE methods.

## Figures and Tables

**Table 1:** Average CIEDE2000 and entropy after applying the UCE and the proposed scheme.

Image Set	Scheme	CIEDE2000				Entropy			
		Original	*S* = 20	*S* = 30	*S* = 40	Original	*S* = 20	*S* = 30	*S* = 40
USC	UCE [28]	0	6.59	9.51	11.40	7.154	7.614	7.709	7.755
	Proposed		4.20	5.99	7.06		7.455	7.513	7.547
Kodak	UCE [28]	0	6.89	9.16	10.71	7.104	7.487	7.562	7.604
	Proposed		4.13	5.58	6.60		7.414	7.492	7.534

**Table 2:** Contrast Enhancement results on 3 test images with *S* = 50.

Test Image	Scheme	RCE	RMBE	SSIM	PSNR	Entropy (Original)	CIEDE 2000
Figure 3a	[28]	0.548	0.933	0.843	19.47	7.788 (7.298)	11.02
(*S* = 50)	Proposed	0.529	0.943	0.859	20.10	7.537 (7.298)	8.98
Figure 3e	[28]	0.485	0.914	0.843	19.74	7.645 (7.293)	13.71
(*S* = 50)	Proposed	0.493	0.961	0.952	24.58	7.496 (7.293)	4.30
Figure 3i	[28]	0.552	0.919	0.862	18.45	7.815 (7.368)	14.00
(*S* = 50)	Proposed	0.549	0.996	0.905	22.41	7.642 (7.368)	6.10

**Table 3:** Statistical comparisons (with a *t*-test) between the UCE and the proposed method.

Metric	Image Set	*t*-Value		
		*S* = 20	*S* = 30	*S* = 40
CIEDE2000	USC (6 images)	4.648	3.011	3.539
	Kodak (24 images)	10.260	9.960	9.155
entropy	USC (6 images)	2.448	2.661	2.694
	Kodak (24 images)	2.688	2.270	2.188

**Table 4:** Statistics (Mean) of contract enhancement effects on two image sets.

*S*	Scheme	USC Image Set				Kodak Image Set			
		RCE	RMBE	SSIM	PSNR	RCE	RMBE	SSIM	PSNR
20	UCE [28]	0.529	0.967	0.918	24.80	0.5348	0.9747	0.9207	24.51
	Proposed	0.517	0.969	0.944	26.31	0.5319	0.9869	0.9367	26.26
30	UCE [28]	0.539	0.954	0.872	21.79	0.5476	0.9624	0.8815	21.79
	Proposed	0.524	0.953	0.910	23.32	0.5445	0.9798	0.8994	23.44
40	UCE [28]	0.545	0.937	0.840	20.13	0.5569	0.9492	0.8540	20.12
	Proposed	0.528	0.941	0.887	21.88	0.5533	0.9738	0.8714	21.80

**Table 5:** Statistics (mean) of recovering original color images.

*S*	Image Set	USC Image Set				Kodak Image Set			
		SSIM	PSNR	Entropy	CIEDE2000	SSIM	PSNR	Entropy	CIEDE2000
20	original	1.0000	∞	7.154	0.0000	1.0000	∞	7.104	0.0000
	recovered	0.9997	60.97	7.153	0.0703	0.9990	54.61	7.101	0.0885
30	original	1.0000	∞	7.154	0.0000	1.0000	∞	7.104	0.0000
	recovered	0.9995	58.86	7.151	0.1016	0.9989	54.40	7.100	0.1092
40	original	1.0000	∞	7.154	0.0000	1.0000	∞	7.104	0.0000
	recovered	0.9994	58.00	7.150	0.1199	0.9989	54.06	7.100	0.1164
